# Accuracy of the Fischer scoring system and the Breast Imaging Reporting and Data System in identification of malignant breast lesions

**DOI:** 10.4103/0256-4947.55310

**Published:** 2009

**Authors:** Hanaa Al-Khawari, Reji Athyal, Agnes Kovacs, Mervat Al-Saleh, John Patrick Madda

**Affiliations:** a Department of Radiology, Faculty of Medicine, Kuwait University, Kuwait; b Department of Radiology, Al-Amiri Hospital, Kuwait; c Department of Surgery, Faculty of Medicine, Kuwait University, Kuwait; d Department of Pathology, Al-Amiri Hospital, Kuwait

## Abstract

**BACKGROUND AND OBJECTIVES:**

Fischer developed a scoring system in 1999 that made identifying malignant lesions much easier for inexperienced radiologists. Our study was performed to assess whether this scoring system would help beginners to accurately diagnose breast lesions on magnetic resonance (MR) imaging and to assess the correlation between the magnetic resonance mammography Breast Imaging Reporting and Data System (MRM BI-RADS) grade and the final diagnosis.

**PATIENTS AND METHODS:**

The lesion morphology and contrast kinetics of 63 masses in 41 patients were evaluated on MRI and accorded a MRM BI-RADS final assessment category using the Fischer scoring system. The accuracy was evaluated after the final diagnosis was obtained by tissue sampling and follow-up imaging.

**RESULTS:**

There were 25 malignant and 30 benign lesions. Eight lesions were seen by MRI only and we could not verify their pathology since we did not have MR-guided biopsy facilities at the time of the study. On MR mammography, the proven carcinomatous lesions were characterized as BI-RADS category V in 16 (64%), category IV in 7 (28%), and category III in 2 (8%) lesions. Benign lesions were graded as category V in 3 (10%), category IV in 6 (20%), and category III in 3 (10%), category II in 10 (33%) and category I in 8 (27%) lesions. The MRM BI-RADS category accurately predicted malignancy in 92% and a benign pathology in 70% of the lesions. The overlap between the MRM features of chronic inflammatory lesions and carcinomas resulted in a lower accuracy in diagnosing benign as compared to malignant lesions.

**CONCLUSION:**

The MRM BI-RADS lexicon using the Fischer scoring system is useful and has a high predictive value, especially for malignant breast lesions, and is easy to apply. Overlapping features between benign inflammatory and malignant lesions might yield a reduced accuracy in inflammatory pathologies.

Breast magnetic resonance imaging (MRI) is extremely sensitive in the detection of carcinoma, with a sensitivity ranging from 97% to 100%.^1^ The specificity is somewhat lower, ranging from 50% to 80% as reported previously.^2^,^3^ The wide range of specificity may be due to the fact that investigators at different institutions use a variety of MR imaging techniques for acquiring and processing images and possibly use different criteria for interpretation. This may also be partly due to differing levels of experience with MR mammography. Given these limitations when interpreting a study, it is important for a radiologist to determine which lesions are suspicious of malignancy and need to be biopsied. A standardized terminology has been developed to facilitate the interpretation and communication among physicians in the description of morphologic findings on mammography. The Breast Imaging Reporting and Data System (BI-RADS),^4^ in the form of a lexicon, that is widely used in the United States, was implemented in our hospital a few years back. It is now the standard for reporting conventional x-ray mammograms and uses descriptors to attach a level of suspicion to a lesion and its need for a biopsy. The same lexicon can be used for reporting MRI breast.^4^

The use of morphologic criteria has been shown to improve specificity when used to describe breast findings on MRI.^5^,^6^ For example, smooth or lobulated margins on MRI show a 97% to 100% predictive value for benignity, whereas the presence of rim enhancement shows a 79% to 92% predictive value for malignancy.^5^ Despite these encouraging results, the value of morphologic criteria to describe MRI-detected breast lesions has been limited by the lack of a definitive classification scheme.

In 1999, Fischer et al^1^ developed a scoring system to predict the likelihood of a lesion on MRI as being benign or malignant. After the magnetic resonance mammography (MRM) BI-RADS classification system (that mirrors the mammographic BI-RADS categories) was described in 2003 by the American College of Radiologists (ACR),^4^ we found that the Fischer scoring system made identifying the BI-RADS category much easier for inexperienced radiologists. Our study was performed to assess whether this scoring system would help beginners to accurately diagnose breast lesions on MR imaging and to assess the correlation between the MRM BI-RADS grade and the final diagnosis.

## PATIENTS AND METHODS

In this single center prospective study conducted in a breast unit of a general hospital, 41 consecutive cases with identified breast lesions on mammography presenting during the period May 2005 to September 2006 underwent an initial dynamic MR mammography of the breast with follow-up imaging studies as required up to 2 years. The study was conducted using a 1.5T magnet (GE Excite, South Carolina, USA) and the patients constituted part of our initial experience with MR mammography.^7^

The patients included in our study were referred to our MR imaging suite with breast lesions for the following reasons:

1. Equivocal findings on mammography (n=18),
2. Suspicion of local relapse in treated breast cancer patients (n=6),
3. Search for a primary breast cancer in patients with metastatic axillary lymph nodes with suspicious lesion on mammography (n=5),
4. Local staging of breast cancer (suspicion of multiple lesions at standard imaging, dense breasts) (n=5),
5. Differentiation between inflammatory benign lesions vs. inflammatory carcinoma (n=4),
6. MRI screening in high-risk patients with lesion suspicious for cancer on mammography (n=3).

We analyzed the morphologic characteristics, enhancement pattern and kinetic features of the lesions and assigned an appropriate final assessment BI-RADS category (Table 1). The aim of the study was to assess whether the descriptive terminology and final assessment categories of the BI-RADS lexicon using the Fischer scoring system^1^ corresponded to the final diagnosis of the lesions as obtained from histopathological confirmation or follow-up.

**Table 1:** Criteria for evaluating breast lesions on contrast-enhanced MR images (the evaluation score).

	Criterion	Points
**1**	**Shape of contrast-enhanced lesion**	
	Round, oval, linear, lobular	0
	Branching, spiculated, stellate	1

**2**	**Margin of contrast-enhanced lesion**	
	Well-defined	0
	Indistinca/ill-defined	1

**3**	**Enhancing pattern of the lesion**	
	Homogenous, non-enhancing internal septations	0
	In-homogenous	1
	Ring enhancement	2

**4**	**Initial signal intensity increase^a^**	
	Less than 50%	0
	50% to 100%	1
	More than 100%	2

**5**	**Signal intensity at 3-6 minutes after contrast injection**	
	Steady increase or continuous^b^	0
	Plateau^c^	1
	Washout^d^	2

a Initial peak signal intensity increase within the first 3 minutes after contrast material administration relative to the precontrast signal intensity.

b Continued signal intensity increase=signal intensity increase of greater than 10% relative to the initial peak signal intensity at 3-8 minutes.

c Plateau=deviation of the signal intensity at 3-8 minutes of (10%) relative to the initial peak signal intensity.

d Washout=signal intensity decrease of greater than 10% relative to the initial peak signal intensity at 3-8 minutes.

### Patient preparation and positioning

A 20-gauge intravenous line was inserted at the dorsum of the hand before positioning the patient on the table to ensure lack of movement between scans. The MRI breast was performed with the patient lying in a prone position on a platform placed in the MR imager that allows the breasts to be in a dependent position. A dedicated 4-channel breast coil was used. MRI was performed during days 6-16 of the menstrual cycle or after stopping hormone replacement therapy for 4-6 weeks to avoid false positive enhancing lesions during the peak hormonal level of the cycle. If the MRI was requested for suspected recurrence of malignancy, the MRI study was performed 6-8 weeks post surgery and at least 9 months (preferably 12 months) post radiotherapy.

### The protocol

Axial T1W 3D SPGR volume images (slice thickness 4 mm, 44 loc per slab, FOV 30, matrix 416×256, NEX 1, flip angle 35, bandwidth 41.67) and sagittal fat-saturated T2W 2D FRFSE images (slice thickness 5 mm, space 1 mm, FOV 36, matrix 320×224, NEX 3, TE 85, TR 5625, echo train length 16, bandwidth 22.73) were obtained prior to contrast injection. Either sagittal or axial fat-saturated T1W high-resolution 3D SPGR dynamic images using vibrant software (slice thickness 4 mm, 66 loc per slab, matrix 256×160, NEX 1, flip angle 12, bandwidth 31.25, ZIP 2 with effective slice thickness of 2.0 mm) were then obtained with a scan time not exceeding 60 sec per scan. Seven acquisitions were obtained simultaneously of both breasts. The first acquisition was obtained before contrast was injected and was used as a mask and the other 6 acquisitions were obtained following contrast injection. The post-contrast acquisitions were finished within 6 minutes. The contrast used was 0.1 mmol/kg of gadolinium-DTPA intravenously, usually injected as a bolus using an automatic injector at a rate of 2mL/sec, followed by a saline flush. The last sequence obtained post contrast was a high T1W spatial resolution sequence; axial 3D SPGR volume fat saturated T1 (slice thickness 4 mm, 44 loc per slab, FOV 30, matrix 416×256, NEX 1, flip angle 35, bandwidth 41.67) which was done at almost 7-8 minutes post contrast.

### Image interpretation

The morphology of the lesion was studied on the 3D volume T1W images, the fat saturated post contrast 3D volume T1W images and the fat saturated T2W images and images obtained post-processing (maximum intensity projection and maximum slope of signal increase). The form, margin and the enhancement pattern of the lesions were identified and scored points as in Table 1. Evaluation of enhancement kinetics following contrast agent administration was done on the post-processed dynamic images using the software function tool 2.6.4b3 of GE to measure the signal enhancement ratio within the lesions and identify the enhancement curves. Since accurate placement of a region of interest over the areas of most rapid and intense enhancement is critical we used color mapping of the lesions as a guide. We followed the quantitative method described by Fischer et al^1^ to study the enhancement curves.

### Evaluating the enhancement curves based on Fischer's group

The evaluation criterion was the peak percentage of signal intensity increase within the first 3 minutes after contrast material administration relative to the precontrast signal intensity (initial signal intensity increase). The initial signal intensity increase within 3 minutes was given a value of less than 50%, 50% to 100% and more than 100%. Furthermore, we evaluated the behavior of the signal intensity curve from the 3rd to the 7th minute. A signal intensity increase of more than 10% within this interval relative to the peak enhancement in the first 3 minutes was defined as “continued signal intensity increase” (giving a type I curve). A signal intensity similar to the peak signal intensity was considered as a plateau (giving a type II curve), and a decrease of more than 10% was defined as a washout (giving a type III curve).

### The Fischer scoring system

**Figure 1:**
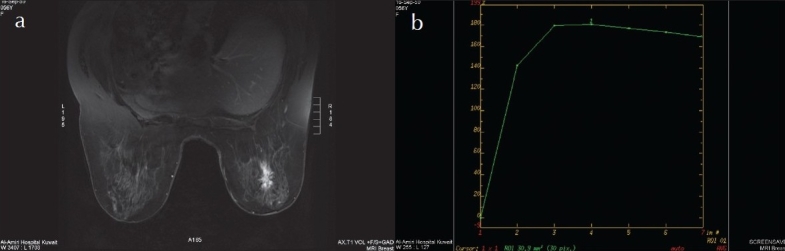
Left breast spiculated mass with ill-defined margin heterogeneous enhancement post contrast with strong initial signal increase and post-initial plateau (type II curve): a) post contrast fat saturated axial T1W image; b) enhancement curve (plateau). Spiculated mass (1 point), ill-defined margin (1 point), heterogeneous enhancement post contrast (1 point), strong initial signal increase (2 points) and post-initial washout (1 point): score 6 points, i.e. MRM BI-RADS category V. Proved ductal carcinoma postoperatively.

**Figure 2:**
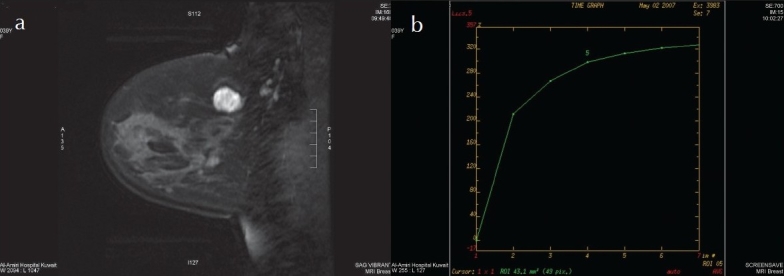
Left breast rounded mass with well-defined margin showing non-enhancing internal septations with strong initial signal increase, and continued signal intensity increase (type 1 curve): a) one minute post contrast fat saturated sagittal image; b) enhancement curve. rounded mass (0 point), well-defined margin (0 point), non-enhancing internal septations (0 point), strong initial signal increase (2 points), continued signal intensity increase (0 point): score 2 points, i.e. MRM BI-RADS category 2. Biopsy proven fibroadenoma.

**Figure 3:**
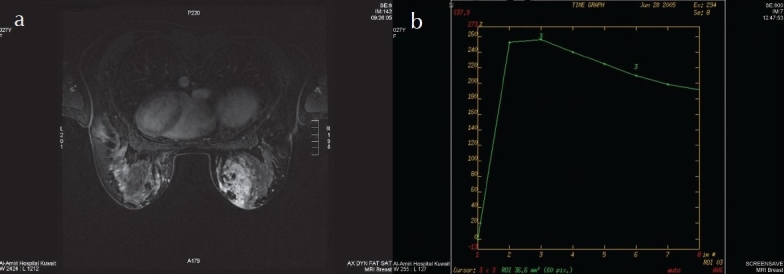
Right breast ill-defined, heterogeneously enhancing oval shaped mass with strong initial signal increase and post-initial washout (type III curve): a) post contrast fat saturated T1W image, b)enhancement curve (washout). Ill-defined mass (1 point), heterogeneously enhancing (1 point), oval shaped mass (0 point), strong initial signal increase (2 points), post-initial washout (3 points): score 7 points, i.e. the MRM BI-RADS category is 5. Histopathology proven chronic granulamatous mastitis.

In this scoring system, five dynamic contrast-enhanced MR features are evaluated. These comprised three morphological (shape, margins, enhancement kinetic) and two functional features (initial peak of signal intensity increase and behaviour of signal intensity curve) constituting a multifactorial protocol in which each criterion receives a point value. Each parameter is assigned points ranging from 0 to 1 or 0 to 2, with higher points for those that are more likely to be associated with malignancy. The sum of all the points defines the degree of suspicion of malignancy, with a score 0 representing the lowest and 8 the highest degree of suspicion (Table 1). The points are then assigned a BI-RAD category); 0 and 1 point correspond to category I (negative, no abnormal enhancement, enhancing masses or architectural distortion, 2 points corresponds to category II (benign findings, for routine follow up), 3 points correspond to category III (probably benign requires short interval follow up after 6 months), 4 and 5 points corresponds to category IV (suspicious abnormality, biopsy should be considered) and 6, 7 and 8 points correspond to category V (highly suggestive of malignancy, biopsy mandatory). The appropriate points for each lesion were thus given to each feature and were recorded in the evaluation score table (Table 1). The BI-RADS category for the lesion was then identified (Figures 1–3. Tissue sampling was performed for cases with BI-RADS categories III, VI and V.

For the sampled lesions, the BI-RADS category was then compared with the tissue sampling result (by FNAC, core biopsy or lumpectomy/ mastectomy histopathology of the tumor). For non-sampled lesions, the BI-RADS category was determined by clinical follow up and repetition of MRI, ultrasonography (US) and/or mammography imaging at 12 and 24 months of the initial MRI scan. We then evaluated the accuracy of BI-RADS category using the Fischer scoring system for both benign and malignant lesions.

## RESULTS

Forty-one patients with identified breast lesions on mammography and ultrasonography were scanned with MRI using the 3D dynamic MRI protocol during May 2005 to September 2006. The age range of patients was 25 to 64 years with a mean age of 44.5 years and a median age of 44 years.

The total number of lesions identified in the 41 patients was 70. Seven of the 70 lesions were seen only on MRI and hence were not proven by tissue sampling since we had not yet started MRI-guided breast biopsy at that time and these were excluded from our study. Of the 63 lesions included in the study, 55 lesions were proved by tissue sampling. Twenty-five of the lesions were malignant and 30 were proven to be benign. Eight of the 63 lesions diagnosed by MRI as benign were not biopsied and were followed-up clinically and radiologically at 12 and 24 months after the initial MRI.

The histopathology of the 25 malignant lesions were well differentiated ductal carcinoma in situ in 5, poorly differentiated infiltrating ductal carcinomas in 8, well-differentiated invasive ductal carcinoma in 6, well-differentiated infiltrating lobular carcinomas in 5, and one well-differentiated mucinous carcinoma. Of the total breast lesions studied, the diagnostic yield of MRI was accurate in detecting malignant lesions in 92% and of benign lesions in 70%. Using the final histopathological diagnosis of the lesions as the gold standard, the sensitivity, specificity, positive and negative predictive values of MRI in diagnosing malignant breast lesions were 96%, 67%, 71% and 95%, respectively.

### Morphological features of 25 pathologically proven malignant lesions

The margins were described as indistinct in 14 (56%) (Figure 1) and well circumscribed in 11 (44%). The shape was described as spiculated in 9 (36%) (Figure 1), round in 8 (32%) branching in 7 (28%) and lobular in 1 (4%). The enhancement pattern was homogeneous in 9 (36%), heterogeneous in 7 (28%) (Figure 1), peripheral rim in 8 (32%) and septated in 1 (4%). The mean size of the 25 carcinomas was 1.6 cm (range, 0.6 to 2.7 cm) for the homogeneously enhancing masses, 2.7 cm (range, 1.0 to 4.4 cm) for the heterogeneously enhancing masses, and 2.7 cm (range, 1.1 to 4.3 cm) for the rim-en-hancing masses. Carcinomas less than or equal to 1 cm were significantly more common in the homogeneously enhancing group than in the heterogeneously or rim en-hancing groups. Of the 25 carcinomas, 10 (40%) were less than 1 cm.

### Morphological features of 30 pathologically proven benign lesions

The margins were described as well circumscribed in 25 (83%) (Figure 2) and indistinct in 5 (17%) masses. The shape was described as round in 14 (47%), branching in 8 (27%), linear in 4 (13%) and spiculated in 1 (3%). The enhancement pattern was homogeneous in 16 (53%), heterogeneous in 8 (27%), and with nonenhancing internal septations in 4 (13%) (Figure 2). The benign lesions that showed irregular or spiculated margin, branching and heterogeneous enhancement were proven to be chronic inflammation (Figure 3). The mean size was 2.2 cm (range, 0.5 to 4.0 cm) for the homogeneously enhancing masses, 3.2 cm (range, 0.3 to 6.0 cm) for the heterogeneously enhancing masses and 1.8 cm for the septated masses (range, 1.2 to 2.4 cm).

### BI-RADS category of both benign and malignant lesions

The BI-RADS classification of the studied 63 lesions was as follows: 19 lesions were classified as BI-RADS V, 13 lesions as BI-RADS IV, 5 lesions as BI-RADS III, 14 lesions as BI-RADS II and 12 lesions as BI-RADS I. Among the 19 lesions classified as BI-RADS V, tissue sampling proved the lesions to be malignant in 16 masses and chronic inflammation in 3 lesions. Among the 13 lesions classified as BI-RADS IV, the lesions were proven by tissue sampling as malignant in 7 lesions, chronic inflammation in 3, complicated cyst in 1 and benign scar tissue in 2. Among the 5 lesions classified as BI-RADS III, 2 lesions were proven malignant and 3 lesions as benign by tissue sampling. Among the 14 lesions classified as BI-RADS II, 10 lesions were proven to be benign by tissue sampling and 4 lesions were proven benign by 2 years clinical and radiological follow up. Among the 12 lesions classified as BI-RADS I, 8 lesions were proven benign by tissue sampling and 4 lesions were proven benign by 2 years clinical and radiological follow-up. Eight lesions were not sampled since we did not started MR-guided breast biopsy at the time of the study. For the benign BI-RADS categories (I and II), the impression on MR mammography was consistent with the final diagnosis of a benign lesions in 100% of the cases. For the malignant MRM BI-RADS category (V), this was found to be consistent with the final diagnosis of malignancy in 84% of the lesions and of MRM BI-RADS category (IV) in 54% of the lesions. Thirty percent (9 out of 30) of benign lesions were diagnosed as malignant or probably malignant. No benign lesion was said to be of a category above II although in one case diagnosed morphologically as fibroadenoma and having BIRADS category III, the final diagnosis was invasive ductal carcinoma. The MRM BI-RADS category was consistent with the histological diagnosis of the 25 malignant lesions in 92% and of the 30 benign lesions in 70% of the masses. The overlapping features of chronic inflammatory lesions with carcinomas (Table 2, Figure 3) was the reason that the MRM BI-RADS category was of lower accuracy in diagnosing the benign lesions as compared to the malignant lesions.

**Table 2:** The morphological features and kinetics of the proved benign lesions with BI-RADS IV and V categories.

	Category	Form	Margin	Enhancing pattern	Initial signal increase	Post-initial course
Lesion 1 Complicated cyst	BI-RADS IV	Branching			Strong	Plateau
Lesion 2 benign	BI-RADS IV	Branching		Inhomogeneous	Strong	
Lesion 3 Chronic inflammation	BI-RADS IV		Indistinct	Inhomogeneous	Strong	
Lesion 4 benign	BI-RADS IV		Indistinct	Inhomogeneous	Strong	
Lesion 5 organizing inflammation	BI-RADS IV	Branching		Inhomogeneous	Strong	Plateau
Lesion 6 Scar tissue	BI-RADS IV				Strong	Washout
Lesion 7 Chronic inflammation	BI-RADS V	Spiculated	Indistinct	Inhomogeneous	Strong	Plateau
Lesion 8 Granulamatous inflammation	BI-RADS V	Branching	Indistinct	Inhomogeneous	Strong	Washout
Lesion 9 Granulamatous inflammation	BI-RADS V	Branching	Indistinct	Inhomogeneous	Strong	Washout

## DISCUSSION

In our study, we found that the Fischer scoring system^1^ and the BI-RADS categories worked well for findings seen on MR imaging. For the 25 malignant lesions, only 2 masses were described as probably benign (BI-RADS category III). Of these one mass showed homogeneous enhancement and the other mass showed heterogeneous enhancement. Both showed circumscribed margins; one was round and the other was lobular in shape. The sizes of both masses were small (10 mm and 20 mm). All the descriptors used in these 2 cases are associated with a benign finding (category II) or probably benign finding (category III) in the final assessment categories. The reason behind this could be due to multiple factors, including limited spatial resolution of the dynamic sequences used and the overlap of enhancement, both in terms of kinetic measurements and morphologic appearances of benign and malignant lesions, which precluded complete differentiation of lesions.^8^–^11^ If these small masses had not been biopsied, a 6-month follow-up would have been performed. As with mammography, if a change were noted at that time, biopsy was performed.

It is becoming increasingly clear that while most investigators have used either enhancement kinetics or lesion morphology in an attempt to differentiate benign enhancing lesions from enhancing breast cancer, an integrated interpretation strategy where enhancement kinetics data and morphologic feature analysis are used together for image interpretation may be superior to the use of either method alone.^12^ Further, since reader variability remains a concern, an imaging lexicon similar to the BI-RADS lexicon used in conventional x-rays mammography, in which the architectural features are defined and illustrated, is needed.

To characterize a lesion as benign or malignant, one should integrate the morphological and the dynamic features of a lesion. The way to do it varies according to the experience of the investigator. For experienced radiologists, the classification of lesions can be done without the need of the scoring system suggested by Fischer et al.^1^ In this case the classification of the lesion is done based on obvious features, e.g. BI-RADS V will be given to a stellate shape or spiculated border irrespective of the enhancement kinetics^13^,^14^ and for other lesions with irregular shape and non-smooth borders and heterogeneous architecture the kinetics are referred to; if there is strong enhancement and washout it is classified as BI-RADS V and if intermediate enhancement with plateau or persistent time course it will then be classified as BI-RADS IV. Classifying a lesion using this method works very well for experienced radiologists, but for inexperienced radiologists this might be confusing and might cause over- or underestimation of the BI-RADS category of a lesion.

Evaluating the enhancement curves can be done with either a qualitative^11^ or quantitative method.^1^ Kuhl et al^8^ described three types of time-intensity curves: type I (steady enhancement), where a persistent increase in signal intensity is present beyond 2 minutes after contrast agent injection; type II (plateau), where the maximum signal intensity is achieved in the first 2 minutes and then remains fairly constant; and type III (wash-out), where the maximum signal intensity is achieved in the first 2 minutes and then decreases over time. It has been reported that the type I curve is usually seen in benign lesions and normal breast parenchyma, the type III curve is mostly seen in malignant lesions and some fibroadenomas and the type II curve is equivocal and seen in some benign lesions and many malignant lesions.^8^ The qualitative method needs experience and is prone to under or overestimating the curve pattern especially in deciding whether it is a plateau (type II curve) or a wash-out (type III curve) pattern. The quantitative method, however, is easier and gives the inexperienced reader more confidence in characterizing the enhancement curve of a lesion.

Non-enhancing internal septations, a descriptor usually associated with fibroadenomas, is a sign that is no longer exclusive to benign lesions since recent results of a study by Schnall et al^11^ revealed that 47% of malignant lesions were shown to have nonenhancing internal septa. In our study, one septated mass that was detected and given BI-RADS III category proved to be well differentiated invasive ductal carcinoma. None of the 25 carcinomas was assigned a final assessment of category I or II. Also, most of them were assigned a BI-RADS category IV (28%) or category V (64%), necessitating biopsy even by those radiologists who had no experience interpreting breast MR images.

For the 30 benign lesions the overall accuracy was less (70%). Three lesions were scored as BI-RADS V and 6 lesions as BI-RADS IV. Looking at the pathological diagnosis, morphology and the kinetics of these lesions (Table 2), we found that 5 of these lesions were chronic inflammatory lesions and 1 mass was scar tissue. The morphologic features of these inflammatory lesions were overlapping with those of the malignant lesions (Figure 3). The difficulty in accurately differentiating chronic inflammation from invasive carcinoma was also reported by Rieber et al.^15^ The inclusion of chronic inflammatory lesions in our study is the main reason for a reduced accuracy since MRI is not a good tool for the differentiation of benign from malignancy in such a clinical situation.

In our study, we noticed a higher prevalence of homogenous enhancement than heterogeneous and rim enhancement in malignant lesions. We also noticed that the mean size of carcinomas showing homogeneous enhancement was smaller than the mean size of carcinomas showing rim or heterogeneous enhancement. The reason behind this could be that 40% of the carcinomas in the current series were less than 1 cm in size. Rim enhancement, seen in 33% of the ductal carcinomas and in 60% of the lobular carcinomas in this series, is considered a suspicious morphologic feature.^2^ The case of mucinous carcinoma in this series did not exhibit rim enhancement.

Nunes et al^5^ found that none of the malignant masses had smooth borders, unlike our study in which 44% of the carcinomas had circumscribed borders. Nevertheless, most of these lesions had other more worrisome descriptors, such as heterogeneous or rim enhancement, which could be interpreted as malignancy as previously reported.^13^ Moreover, mammographically circumscribed masses can be malignant, particularly the specific histologic subtypes of medullary, colloid, and papillary carcinoma as 5% to 6% of malignant masses have been described as circumscribed.^16^

In our study, we noted that there was a good correlation between the MRM-BI-RADS final assessment category and the eventual diagnosis obtained on histopathology or follow-up. One weakness of our study was that we were forced to exclude 7 lesions from the study and analysis as they required MR-guided biopsy that we did not perform at the start of the study. It is no doubt important to have the facility of performing biopsies under MR guidance for lesions not accessible by other modalities and in whom a wait and watch policy is not advised.

It is logical that mammographers who are accustomed to evaluating breast lesions using BI-RADS terms on mammography can readily translate those skills to MRI. The assignment of a final assessment category, as in mammography, indicates to the referring physician what appropriate step should be taken next and what information should be included in the report.

In conclusion, the MRM BI-RADS lexicon using the Fischer system is easy to apply and very useful and accurate in characterizing breast lesions. Overlapping MR features between some benign lesions like chronic inflammation and malignancy that might reduce the specificity need to be interpreted in conjunction with the clinical presentation and all other pertinent breast imaging studies, such as mammography and ultrasonography.
